# Multimorbidity and Functional Limitations Among Adults 65 or Older, NHANES 2005–2012

**DOI:** 10.5888/pcd13.160174

**Published:** 2016-11-03

**Authors:** Kazuaki Jindai, Carrie M. Nielson, Beth A. Vorderstrasse, Ana R. Quiñones

**Affiliations:** Author Affiliations: Kazuaki Jindai, School of Public Health, Oregon Health & Science University, Portland, Oregon, and VA Portland Health Care System, Portland, Oregon; Carrie M. Nielson, Beth A. Vorderstrasse, School of Public Health, Oregon Health & Science University, Portland, Oregon. Dr Quiñones is also affiliated with VA Portland Health Care System, Portland, Oregon. Dr Vorderstrasse is now affiliated with Health Promotion and Chronic Disease Prevention, Oregon Health Authority, Portland, Oregon.

## Abstract

**Introduction:**

The development of functional limitations among adults aged 65 or older has profound effects on individual and population resources. Improved understanding of the relationship between functional limitations and co-occurring chronic diseases (multimorbidity) is an emerging area of interest. The objective of this study was to investigate the association between multimorbidity and functional limitations among community-dwelling adults 65 or older in the United States and explore factors that modify this association.

**Methods:**

We conducted a cross-sectional analysis of adults aged 65 or older using data from the National Health and Nutrition Examination Survey (NHANES) from 2005 through 2012. We used negative binomial regression to estimate the association between multimorbidity (≥2 concurrent diseases) and functional limitations and to determine whether the association differed by sex or age.

**Results:**

The prevalence of multimorbidity in this population was 67% (95% confidence interval [CI], 65%–68%). Each additional chronic condition was associated with an increase in the number of functional limitations, and the association was stronger among those aged 75 or older than among those aged 65 to 74. For those aged 65 to 74, each additional chronic condition was associated with 1.35 (95% CI, 1.27–1.43) times the number of functional limitations for men and 1.62 times (95% CI, 1.31–2.02) the number of functional limitations for women. For those 75 or older, the associations increased to 1.71 (95% CI, 1.35–2.16) for men and 2.06 (95% CI, 1.51–2.81) for women for each additional chronic condition.

**Conclusion:**

Multimorbidity was associated with increases in functional limitations, and the associations were stronger among women than among men and among adults aged 75 or older than among those aged 65 to 74. These findings underscore the importance of addressing age and sex differences when formulating prevention strategies.

## Introduction

Older adults are at greatest risk developing chronic illnesses and related functional limitations (1,2). *Healthy People 2020* prioritizes the reduction in the proportion of older adults with moderate to severe functional limitations from 29.3% in 2007 to 26.4% in 2020 (3). Achieving this goal necessitates identifying contributors to loss of function among these adults and using this information to better inform prevention programs.

The association between disability and a high number of chronic diseases is documented (4,5). Often, the etiological pathway from disease to disability is conceptualized as a trajectory from disease pathology to impairment, functional limitation, and finally, to disability. However, this trajectory is modified by personal and environmental circumstances (6,7). Older women, the oldest old, and older adults with low educational attainment report the greatest difficulty with functional capacity (8).

More than 50% of older adults and 70% of Medicare beneficiaries have multimorbidity, defined as having 2 or more co-occurring chronic diseases (9–11). An increase in the number of chronic conditions may contribute to greater functional limitation and disability: 45% of people with chronic conditions have some type of activity limitation (12), and longitudinal studies indicate that an increase in multimorbidity is associated with greater limitations in activities of daily living (13). The US Department of Health and Human Services framework on multiple chronic conditions emphasizes the importance of improving the health and function of those who have co-occurring chronic diseases (14) rather than focusing on individual chronic conditions; this critical shift is designed to ensure effective, holistic care for older adults with multimorbidity (2,15).

The objective of this study was to examine the relationship between multimorbidity and functional limitation in a representative sample of community-dwelling older adults in the United States. We tested the hypothesis that multimorbidity is associated with higher levels of functional limitation and that this association is modified by sex and age.

## Methods

### Data source

We compiled data from 4 waves of the National Health and Nutrition Examination Survey (NHANES) conducted from 2005 through 2012. NHANES is an ongoing national survey that collects information from approximately 10,000 civilian, noninstitutionalized adults and children every 2 years. Unique participants are recruited for each wave; therefore, participants are not followed over time. Data on participants’ demographic characteristics, socioeconomic status, health, and diet are collected through interviews, physical examinations, and laboratory tests. Sampling is performed by using a complex, multistage probability design, and weighting is used in the analysis to generalize to the US population. As a result, NHANES provides comprehensive data on morbidity and sociodemographic characteristics for a large, nationally representative sample of adults and is an appropriate data set for assessing national prevalence estimates of multimorbidity and the association with functional limitations. Additional details on NHANES methods and data collection are described elsewhere (16). Because this study involved secondary data analysis of publicly available and de-identified data, the institutional review board at Oregon Health & Science University considered it exempt from human subjects review.

### Participants

NHANES participants were included in this study if they were aged 65 years or older and completed questionnaires relevant to all study variables, including patient health questionnaires at the NHANES mobile examination centers. Of the 40,790 people included in the 4 waves of NHANES, 5,518 people were aged 65 or older. Among these, 4,555 completed all relevant questionnaire items and had data (obtained at mobile examination centers) on height and weight, which were used to calculate body mass index (BMI). We did not have complete data on 963 of the 5,518 participants who met the age criterion. Excluded participants had missing data on one or more components of the limitation score, one or more of the 9 chronic conditions that formed the aggregate multimorbidity score, or missing data for BMI.

### Measures

Comparable variables across all 4 waves were included in the study. Our primary outcome of interest was the number of functional limitations, which was derived from the 19 NHANES questionnaire items used to assess functional status (Box). These questions assess a person’s ability to perform tasks without the use of any special equipment and are organized into 5 major domains: 1) activities of daily living (ADL), 2) instrumental activities of daily living (IADL), 3) leisure and social activities, 4) lower-extremity mobility, and 5) general physical activities. For this analysis, the domains were combined and treated as one construct to provide an aggregate count of functional limitations ranging from 0 to 19 (17). We used the term “functional limitations” to assess a person’s ability to perform a wide range of physical and mental activities; in contrast, the term “disability” usually refers to difficulty performing activities that are seen as necessary to engage in everyday life (13). Responses to each of the 19 questions were coded as 0 if the participant reported performing an activity with no difficulty; responses were coded as 1 if the participant reported some difficulty, much difficulty, or “unable to do.” The percentage of responses of “do not do this activity” ranged from 0.07% (for dressing) to 5% (for preparing meals); we treated those responses as missing data, which was also done in previous studies using NHANES data (18). For the 2 questions on lower-extremity mobility, responses were coded as 1 if the participant indicated a need for specialized equipment for walking in a preceding gateway question.

**Box. NHANES Questionnaire Items (19 Activities in 5 Domains) Used to Assess Functional Status**

**Domain: Activities of daily living**

Getting in and out of bed

Using fork, knife, drinking from cup

Walking between rooms on same floor

Dressing yourself

**Domain: Instrumental activities of daily living**

House chores

Managing money

Preparing meals

**Domain: Leisure and social activities**

Going out to movies, events

Leisure activity at home

Attending social events

**Domain: Lower extremity mobility**

Walking up 10 steps

Walking for a quarter-mile

**Domain: General physical activities**

Grasping or holding small objects

Lifting or carrying

Reaching up over head

Sitting for long periods

Standing for long periods

Standing up from armless chair

Stooping, crouching, kneeling

The primary exposure of interest in this study was multimorbidity. NHANES collects self-reports of 9 health conditions by asking whether the participant has “ever been told by a doctor or health professional” that he or she has the health condition. For this study, multimorbidity was constructed as a sum of responses to having received a diagnosis of any of 9 chronic conditions asked about in NHANES: arthritis, cancer, cardiovascular disease (congestive heart failure, coronary heart disease, or angina), chronic kidney disease, depression, diabetes, hypertension, pulmonary disease (emphysema, chronic bronchitis, or asthma), and stroke (9,19). Depression was dichotomized as a score of less than 10 or a score of 10 or more on the 9-item Patient Health Questionnaire. Scores of 10 or more have high sensitivity and specificity for identifying major depression in a primary care setting (20). We explored differences between men and women in the prevalence of these 9 chronic conditions by comparing and testing for significant differences.

Covariates that could confound the relationship between multimorbidity and functional limitations are age, smoking, BMI, income, race/ethnicity, and education level; these covariates were identified in previous research (5,9,21). In addition, studies of older adults identified sex and marital status as effect modifiers (1,22). We operationalized covariates as follows: 1) because we found few study participants at the high end of the age range (only 97 participants were aged ≥85 y), we categorized age into 2 groups: a younger age group (65–74 y) or an older age group (≥75 y); 2) smoking status was a categorical variable (never, former, or current); 3) BMI was calculated by using measured height and weight with the conventional formula (weight [in kg] divided by height [in m^2^]); 4) annual household income was a categorical variable calculated as a percentage of the federal poverty level (FPL) (<100% FPL, >100% FPL but <200% FPL, or ≥200% FPL); 5) race/ethnicity was categorized as non-Hispanic white, non-Hispanic black, Hispanic, or other; 6) education was categorized as less than 9th grade, at least 9th grade but less than 12th grade, high school graduate or general educational development (GED), some college or associate degree, and college graduate or higher; 7) sex was dichotomized as female or male; 8) marital status was dichotomized as either married/living with a partner or single/other. We also included a binary variable for having a usual source of health care to account for possible underreporting of morbidity through lack of access to a diagnosis.

### Statistical analysis

The dependent variable, functional limitations, is a count variable that is overly dispersed (ie, many people have no limitations and few people have several limitations). As a result, we conducted negative binomial regression analyses to estimate the association between multimorbidity and functional limitations. These models allowed us to calculate the ratio of the mean number of functional limitations associated with each increase in the number of chronic conditions. We also applied survey analysis weights when estimating population proportions, regression coefficients, and 95% confidence intervals (CIs) (16).

We examined a base model that included multimorbidity, age, BMI, and smoking. These were included a priori because of their theoretically relevant relationships to both chronic disease prevalence and functional limitation. Inclusion of additional covariates was assessed by entering all covariates and removing each variable sequentially to assess the change in the ratio of the mean number of functional limitations from the negative binomial regression. If removal of a covariate changed the ratio of means by more than 10%, it was retained in the model and considered a confounder (23). No additional covariate altered the measure of association by 10%, and so none were included in the final model. Potential interactions between multimorbidity and sex, marital status, and age group (65–74 vs ≥75 y) were tested. The final model included multimorbidity, the interaction term between multimorbidity and sex, and the interaction term between multimorbidity and age group. All statistical analyses were conducted by using Stata version 13 (StataCorp LP).

We conducted sensitivity analyses to assess the robustness of findings to the operationalization of the dependent variable and multimorbidity, the primary exposure of interest. We assessed whether our findings held when operationalizing the functional limitations of ADL and IADL domains only, which are considered to be later manifestations of functional loss than other physical function domains (6,24).

## Results

The mean age of the study population was 73 years, 55.9% were women, and 71.0% were overweight or obese (Table 1). Most (82.4%) participants were non-Hispanic white, and 60.7% were married or living with a partner. Approximately one-third (34.6%) had an annual household income of less than 200% of the FPL. Most participants reported having a usual source of health care (97.4%).

**Table 1:** Characteristics of the Study Sample^a^, National Health and Nutrition Examination Survey, 2005–2012^b^

Characteristic	Value (N = 4,555)
**Age, mean (SE), y**	73.2 (0.1)
**Age group, n (%)**	
65–74 y	2,518 (58.2)
≥75 y	2,037 (41.8)
**Women**	2,254 (55.9)
**Race/ethnicity**	
Non-Hispanic white	2,765 (82.4)
Non-Hispanic black	818 (7.7)
Hispanic	770 (6.3)
Other	202 (3.5)
**Married or living with partner**	2,566 (60.7)
**Annual household income as percentage of federal poverty level, adjusted for location of residence**	
<100%	653 (8.7)
≥100% and <200%	1,356 (25.9)
≥200%	2,546 (65.4)
**Education**	
<9th grade	838 (11.1)
9th to <12th grade	757 (14.1)
High school graduate or equivalent	1,132 (26.9)
Some college or associate degree	1,006 (25.2)
College graduate or above	814 (22.7)
**Has a usual source of health care**	4,395 (97.4)
**Body mass index, kg/m^2^**	
Underweight (<18.5)	68 (1.5)
Normal (≥18.5 and <25.0)	1,219 (27.5)
Overweight (≥25.0 and <30.0)	1,694 (37.0)
Obese (≥30.0)	1,574 (34.0)
**Smoking status**	
Never	2,154 (47.8)
Former	1,937 (43.2)
Current	462 (9.0)

a NHANES participants were included in this study if they were aged 65 years or older and completed questionnaires relevant to all study variables.

b Values are unweighted counts and weighted percentages unless otherwise indicated.

Multimorbidity ranged from 0 to 8 chronic conditions (Table 2). The estimated prevalence of having 2 or more concurrent chronic conditions was 67% overall (95% CI, 65%–68%); it was 64% (95% CI, 61%–66%) among participants aged 65 to 74 and 71% (95% CI, 68%–73%) among those aged 75 or older. The number of limitations ranged from 0 to 19. Overall, 64% of participants had at least 1 functional limitation; the percentage of those who had difficulty performing activities was greater among those aged 75 or older than among those aged 65 to 74 except for 1 activity, sitting for long periods (Appendix). We found a difference between sex and age group; women reported a greater number of limitations than men in both age groups.

**Table 2:** Distribution of Chronic Conditions and Functional Limitations by Age and Sex, National Health and Nutrition Examination Survey, 2005–2012^a^

No. of Conditions or Limitations	Overall	Aged 65–74		Aged ≥75	
		Men	Women	Men	Women
**Chronic conditions**					
0	11 (9–12)	13 (11–15)	12 (9–15)	9 (7–11)	8 (6–10)
1	23 (21–24)	23 (20–27)	24 (21–28)	22 (19–25)	20 (17–23)
2	27 (26–29)	27 (24–31)	27 (24–31)	25 (22–28)	29 (26–33)
3	22 (20–24)	22 (19–25)	22 (18–26)	23 (20–26)	22 (19–25)
4–8	17 (16–19)	15 (12–17)	14 (12–17)	21 (18–24)	21 (18–24)
**Functional limitations^b^**					
0	36 (34–38)	50 (46–54)	36 (32–39)	32 (28–35)	25 (22–28)
1–4	39 (37–40)	33 (30–37)	39 (36–42)	43 (40–46)	42 (38–46)
5–9	14 (13–15)	9 (7–11)	14 (12–17)	14 (11–16)	19 (17–22)
10–19	11 (10–12)	8 (6–10)	11 (9–13)	12 (10–15)	15 (13–17)

a All values are weighted percentage (95% confidence interval).

b χ^2^ for test of differences in functional limitations by sex and age: *P* < .001.

**Figure 1:**
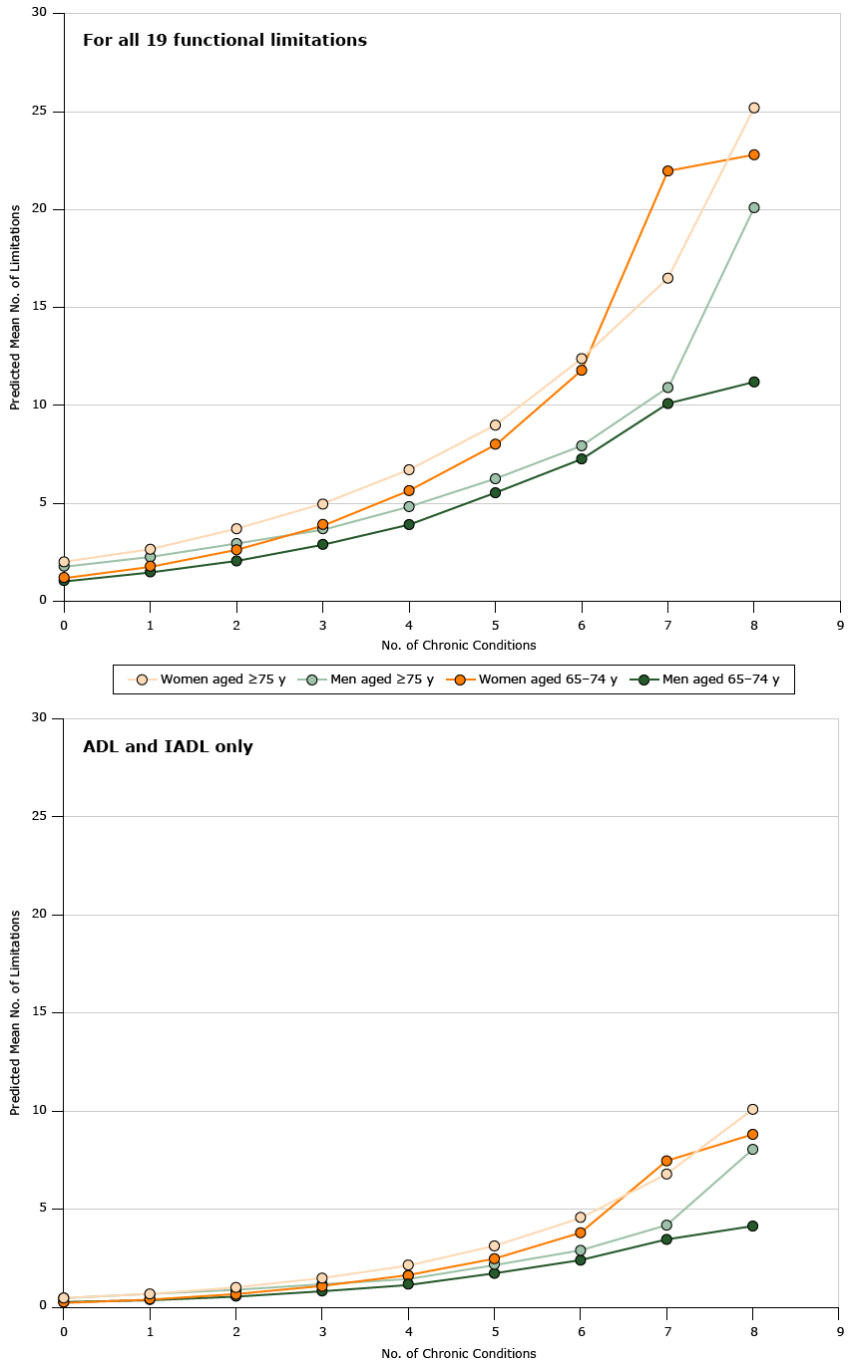
Predicted mean number of limitations by the number of chronic conditions, stratified by sex and age group, National Health and Nutrition Examination Survey 2005–2012, for all 19 limitations and for ADL and IADL (7 limitations) only. Negative binomial regression was used to estimate the association between multimorbidity and functional limitation, adjusted for age, body mass index, and smoking. Abbreviations: ADL, activities of daily living; IADL, instrumental activities of daily living. **Table:** 

We found a significant positive association between multimorbidity and the number of functional limitations and a significant interaction with age (*P* = .02) (Table 3). The magnitude of the association was also stronger among women than among men: for each additional chronic disease, the mean number of limitations among women aged 65 to 74 increased by an estimated 1.62 (95% CI, 1.31–2.02) times and among women aged 75 or older by 2.06 (95% CI, 1.51–2.81) times (Table 3 and Figure 1). In contrast, for each additional chronic disease, the mean number of limitations among men aged 65 to 74 increased by an estimated 1.35 (95% CI, 1.27–1.43) times and among men aged 75 or older by 1.71 (95% CI, 1.35–2.16) times. Associations found for ADL and IADL limitations only were similar to the associations found for all limitations in direction, magnitude, and significance in all age and sex categories (Table 3).

**Table 3:** Associations of Age, BMI, Smoking Status, and Sex With the Number of Functional Limitations Among Adults Aged ≥65 Years, NHANES, 2005–2012

Characteristic	All Limitations^a^		ADL and IADL Limitations Only^b^	
	Ratio of Means^c^ (95% CI)	*P* Value	Ratio of Means^c^ (95% CI)	*P* Value
**Age (per 5-year increase)**	1.14 (1.01–1.28)	.04	1.14 (0.95–1.36)	.15
**BMI (per 5-kg/m^2^ increase)**	1.14 (1.09–1.18)	<.001	1.12 (1.05–1.19)	.001
**Smoking status^a^**				
Never	1 [Reference]		1 [Reference]	
Former	1.01 (0.91–1.11)	.90	0.95 (0.82–1.09)	.46
Current	1.28 (1.05–1.57)	.02	1.15 (0.86–1.52)	.34
**Women**				
No. of chronic conditions for women aged 65–74	1.62 (1.31–2.02)	<.001	1.50 (1.09–2.05)	.01
No. of chronic conditions for women aged ≥75	2.06 (1.51–2.81)	<.001	2.00 (1.23–3.27)	.006
**Men**				
No. of chronic conditions for men aged 65–74	1.35 (1.27–1.43)	<.001	1.41 (1.30–1.54)	<.001
No. of chronic conditions for men aged ≥75	1.71 (1.35–2.16)	<.001	1.89 (1.31–2.71)	.001

Abbreviations: ADL, activities of daily living; BMI, body mass index; CI, confidence interval; IADL, instrumental activities of daily living; NHANES, National Health and Nutrition Examination Survey.

a The 19 NHANES questionnaire items used to assess functional status.

b ADL are getting in and out of bed; using fork, knife, drinking from cup; walking between rooms on same floor; and dressing yourself. IADL are house chores, managing money, and preparing meals.

c Ratio of means is the relative increase in functional limitations associated with each additional chronic condition after adjustment for age, BMI, and smoking status. For example, for women aged 65 to 74, each additional chronic condition is associated with a 62% increase in the mean number of functional limitations after adjustment for age, BMI, and smoking status. Associations of age, BMI, and smoking associations with number of limitations were consistent across sex and age groups. The association with multimorbidity differed by age group (*P*_interaction_ = .02) for all limitations but was not significant for ADL and IADL limitations (*P*_interaction_ = .15). The association with multimorbidity did not differ significantly between sexes (both *P*_interaction_ ≥ .23).

No. of Chronic Conditions	Men		Women	
	Aged 65–74 y	Aged ≥75 y	Aged 65–74 y	Aged ≥75 y
**For all 19 limitations**				
0	1.07	1.80	1.22	2.02
1	1.51	2.27	1.80	2.66
2	2.07	2.96	2.64	3.71
3	2.91	3.71	3.93	4.97
4	3.92	4.84	5.66	6.72
5	5.55	6.26	8.03	9.00
6	7.27	7.94	11.8	12.4
7	10.1	10.9	22.0	16.5
8	11.2	20.1	22.8	25.2
**For ADL and IADL only**				
0	0.28	0.49	0.28	0.49
1	0.41	0.66	0.44	0.70
2	0.58	0.90	0.68	1.03
3	0.85	1.19	1.08	1.50
4	1.20	1.63	1.63	2.17
5	1.74	2.21	2.49	3.14
6	2.41	2.91	3.81	4.59
7	3.47	4.20	7.47	6.80
8	4.15	8.06	8.82	10.1

**Figure 2:**
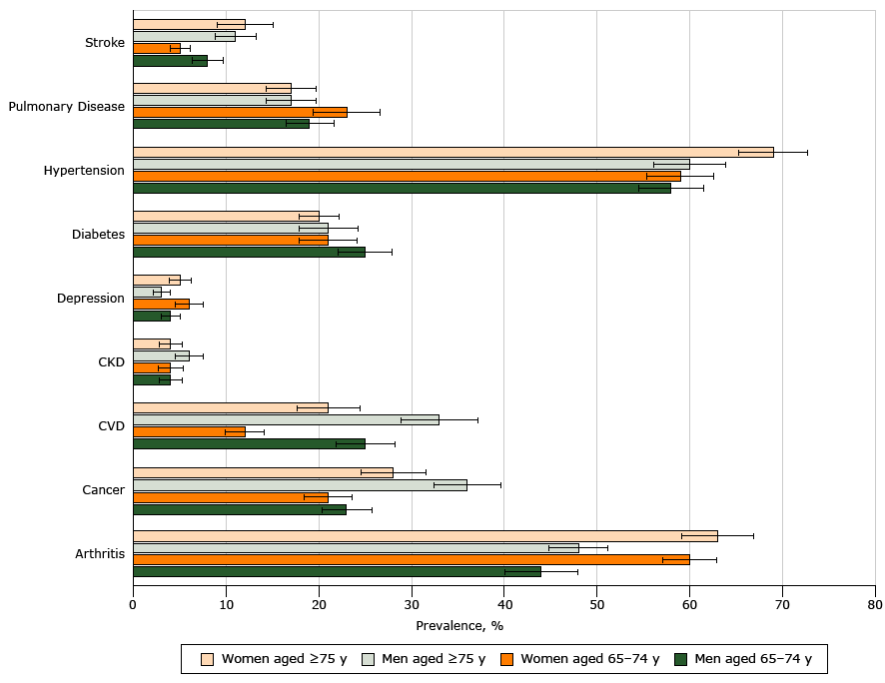
Prevalence of 9 chronic conditions, by age and sex, among adults aged ≥65, National Health and Nutrition Examination Survey, 2005–2012. For pairwise comparisons of prevalence between sexes in each age group and between age groups of each sex, we found the following significant differences using a Bonferroni-corrected *P* value of <.001: between sexes in both age groups for CVD and arthritis; between women’s age groups for CVD; between women’s age groups for stroke and hypertension; between men’s age group for cancer. Error bars are 95% confidence intervals. Abbreviations: CKD, chronic kidney disease; CVD, cardiovascular disease. **Table:** 

Women had a significantly higher prevalence of arthritis than men in both age groups (Figure 2). In contrast, the prevalence of cardiovascular disease was higher for men than women in both age groups. Among women, the prevalence of stroke and hypertension was significantly higher for the older age group than for the younger group; and among men, the prevalence of cancer was significantly higher for the older age group than the younger age group.

Sex and Age	Arthritis	Cancer	Cardiovascular Disease	Chronic Kidney Disease	Depression	Diabetes	Hypertension	Pulmonary Disease	Stroke
Men aged 65–74	44 (40–48)	23 (20–26)	25 (22–29)	4 (3–6)	4 (3–5)	25 (22–28)	58 (54–61)	19 (16–22)	8 (6–10)
Women aged 65–74	60 (57–63)	21 (19–24)	12 (10–14)	4 (3–6)	6 (5–8)	21 (18–25)	59 (55–62)	23 (19–27)	5 (4–6)
Men aged ≥75	48 (45–51)	36 (32–40)	33 (29–37)	6 (4–8)	3 (2–4)	21 (18–25)	60 (56–63)	17 (15–21)	11 (9–14)
Women aged ≥75	63 (59–66)	28 (25–32)	21 (17–24)	4 (3–6)	5 (4–6)	20 (18–23)	69 (65–72)	17 (15–20)	12 (9–16)

In sensitivity analyses, we found that limiting the dependent variable to only the ADL and IADL domains did not substantively alter the magnitude or direction of the association between multimorbidity and functional limitations.

## Discussion

In our study of community-dwelling adults aged 65 or older, we found a significant positive association between multimorbidity and functional limitations. Moreover, the magnitude of the association was significantly higher among women than men, highlighting the possibility that sex influences the relationship between disease burden and functional limitations. These findings are consistent with previous studies that examined the association between self-reported multimorbidity and disability among older adults. Recent studies also found an association between self-reported multimorbidity with functional limitations and disability; they found that increasing levels of multimorbidity were associated with worsening functional capacity (4,5).

Our study found that sex and age modified the association between multimorbidity and function. These findings have implications for practice and policy, suggesting that prevention and self-management programs should be targeted toward older women to delay institutionalization and high-cost care. These findings are consistent with reports of higher levels of disability among older women in Europe (22) and studies using US data showing that women have longer life expectancy than men yet may spend more years in a disabled state (8,25). We also explored the possibility that certain combinations of chronic conditions are more prevalent and associated with more functional limitations for older women relative to their male counterparts (26,27), and we found no differences by sex after we included age group interactions. The interaction between sex and multimorbidity was not significant in our study when we included age groups, probably because of the importance of the onset of age-related multimorbidity in the association between multimorbidity and functional limitations. Hence, we found moderate support for our hypothesis that associations are modified by sex, but advancing age could be a major driver of functional limitations for adults with multimorbidity.

Our study has several limitations. Because only noninstitutionalized people are included in the NHANES study population and because certain measures are collected only for those that can answer independently (without proxy) and travel to the mobile examination centers, our findings are probably generalizable only to adults aged 65 or older who are not severely disabled or cognitively impaired. People with physical conditions or cognitive impairments severe enough to require institutionalization or assistance in answering questions were excluded from the sample population. Our analyses were also limited by the number of diseases that the NHANES survey asked about. Although all 9 diseases asked about in NHANES are on the list of 20 conditions in the US Department of Health and Human Services’ report that seeks to standardize the measurement of multimorbidity (14,28), some diseases that are on the list (eg, dementia, schizophrenia) could not be examined in our study because they are not asked about in NHANES. Still, our findings contribute to the understanding of multimorbidity and sex interactions with functional limitations (5).

The mode in which NHANES collects data on disease status (ie, by self-report instead of physician report) often raises concern about the reliability of the data. Whether or not self-reports yield valid estimates of disease status (29), self-perceptions of illness are key determinants of self-management behavior and are associated with various health outcomes (30). Although clarification of the severity or duration of the conditions that contribute to multimorbidity would be useful, we could not assess these factors because NHANES does not collect this information. These are areas for future research.

Additional, unmeasured factors that putatively influence the relationship between multimorbidity and functional limitations — such as rural or urban residence, or more proximal “get-up-and-go” functional performance measures — could modify our findings on how sex and age influence this relationship. Data on these factors are not readily available in NHANES, however, so we could not include them. These factors should be considered in future studies. Finally, reciprocal linkages probably exist between multimorbidity and functional limitations; these could not be examined in our study because of its cross-sectional design. Studies of longitudinal samples should examine the onset of functional limitations with existing multimorbidity (and vice versa) to assess the temporal relationship.

Those limitations notwithstanding, our study provides insights into the association between multimorbidity and functional limitation among adults aged 65 or older, particularly our finding that the association is stronger among those 75 or older than among those 65 to 74 and among women than among men. Although a causal association cannot be inferred from this cross-sectional study, it is plausible that interventions aimed at reducing chronic disease could reduce functional limitation and preserve independence. Disease, functional limitation, and disability should not be seen as inevitable results of aging and could be mitigated by public health interventions to reduce multimorbidity. The findings of this study also emphasize the need to address age and sex differences in functional limitations associated with multimorbidity and to develop interventions for high-risk adults 65 or older. It will be increasingly important for clinical practice and health care policies to respond to demographic trends in multimorbidity to encourage systemwide efforts to improve chronic disease management for people who have multiple chronic conditions, particularly adults 65 or older.

## Figures and Tables

**Figure:**
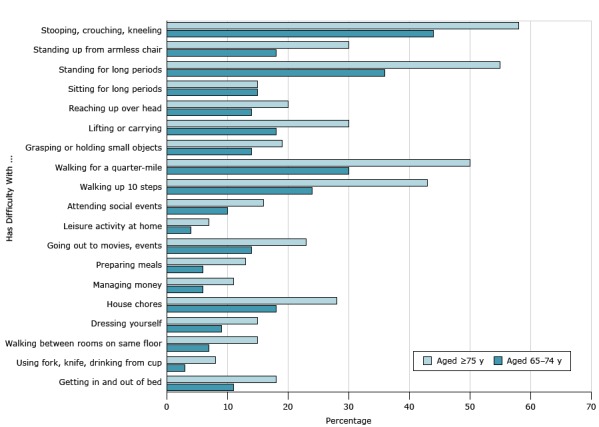
Prevalence among older adults of having difficulty performing 19 activities, by age group, National Health and Nutrition Examination Survey (NHANES), 2005–2012. The list of activities was derived from 19 NHANES questionnaire items, grouped into 5 domains, used to assess functional status. Responses to each question were dichotomized as either having no difficulty or having difficulty (some difficulty, much difficulty, or “unable to do”). For example, if the prevalence among adults aged 65 to 74 of having difficulty getting in and out of bed was 11%, then the prevalence of not having difficulty was 89%. **Table:** 

Domain/Activity	Has Difficulty With Activity	
	Aged 65–74 y	Aged ≥ 75 y
**Activities of daily living**		
Getting in and out of bed	11	18
Using fork, knife, drinking from cup	3	8
Walking between rooms on same floor	7	15
Dressing yourself	9	15
**Instrumental activities of daily living**		
House chores	18	28
Managing money	6	11
Preparing meals	6	13
**Leisure and social activities**		
Going out to movies, events	14	23
Leisure activity at home	4	7
Attending social events	10	16
**Lower-extremity mobility**		
Walking up 10 steps	24	43
Walking for a quarter-mile	30	50
**General physical activities**		
Grasping or holding small objects	14	19
Lifting or carrying	18	30
Reaching up over head	14	20
Sitting for long periods	15	15
Standing for long periods	36	55
Standing up from armless chair	18	30
Stooping, crouching, kneeling	44	58
